# Impact of Disinfection and Sterilization on 3D-Printing Resin Performance for Surgical Guides in Cardiac Ablation Surgery

**DOI:** 10.3390/bioengineering12090924

**Published:** 2025-08-28

**Authors:** Rani Kronenberger, Rawan Kazma, Alireza Amirabadi, Leire Viana Uribe, Giacomo Talevi, Görkem Eylül Kaya, Niko Van den Brande, Ramak Hossein Abadi, Kalliopi-Artemi Kalteremidou, Danny Van Hemelrijck, Kitty Baert, Tom Hauffman, Jeroen Soete, Luigi Pannone, Andrea Maria Paparella, Ivan Eltsov, Gian Battista Chierchia, Mark La Meir, Ali Gharaviri, Carlo de Asmundis

**Affiliations:** 1 Cardiac Surgery Department, Vrije Universiteit Brussel, Universitair Ziekenhuis Brussel, Laarbeeklaan 101, 1050 Brussels, Belgium; mark.lameir@uzbrussel.be; 2 Heart Rhythm Management Centre, Universitair Ziekenhuis Brussel, Heart Rhythm Research Brussels, Postgraduate Program in Cardiac Electrophysiology and Pacing, Vrije Universiteit Brussel, European Reference Networks Guard-Heart, 1090 Brussels, Belgium; rawan.kazma@vub.be (R.K.); alireza.mahdian.amirabadi@vub.be (A.A.); leire.viana.uribe@vub.be (L.V.U.); giacomo.talevi@uzbrussel.be (G.T.); luigi.pannone@uzbrussel.be (L.P.); andrea.paparella@uzbrussel.be (A.M.P.); ivan.eltsov@vub.be (I.E.); jeanbaptiste.chierchia@uzbrussel.be (G.B.C.); ali.gharaviri@uzbrussel.be (A.G.); carlo.deasmundis@uzbrussel.be (C.d.A.); 3 Research Group Sustainable Materials Engineering (SUME)—Lab of Physical Chemistry and Polymer Science (FYSC), Vrije Universiteit Brussel, 1050 Brussels, Belgium; gorkem.eylul.kaya@vub.be (G.E.K.); niko.van.den.brande@vub.be (N.V.d.B.); 4 Department of Mechanics of Materials and Constructions, Vrije Universiteit Brussel, 1050 Brussels, Belgium; ramak.hossein.abadi@vub.be (R.H.A.); kalliopi-artemi.kalteremidou@vub.be (K.-A.K.); danny.van.hemelrijck@vub.be (D.V.H.); 5 Research Group Sustainable Materials Engineering—Lab of Electrochemical and Surface Engineering (SURF), Vrije Universiteit Brussel, 1050 Brussels, Belgium; kitty.baert@vub.be (K.B.); tom.hauffman@vub.be (T.H.); 6 Department of Materials Engineering, KU Leuven, 3001 Leuven, Belgium; jeroen.soete@kuleuven.be

**Keywords:** 3D printing, autoclaving, disinfection, surgical guide, thermal characterization, mechanical properties, vaporized hydrogen peroxide

## Abstract

Patient-tailored, 3D-printed surgical guides offer significant potential to improve precision and therapeutic efficacy in cardiac ablation surgery. However, reliable post-sterilization material performance presents a critical yet underexplored barrier to clinical adoption. This study investigates how disinfection and sterilization impact the mechanical and thermal properties of photopolymer resins. Specimens from two 3D-printing resins (Bioflex A80 MB™, 3Dresyns; MED625FLX™, Stratasys) were treated with four combinations of disinfection techniques (low-temperature manual cleaning; high-temperature machine washing) and sterilization techniques (H_2_O_2_ vs. autoclaving). We assessed post-sterilization properties by mechanical (material integrity, bending tests), thermal (differential scanning calorimetry, thermogravimetric analysis), and viscoelastic (dynamic mechanical analysis) studies. Statistical analysis was performed using one-way ANOVA with Bonferroni post hoc tests (α = 0.05). From this preliminary study, we conclude that MED625FLX maintains integrity and flexibility across all tested disinfection and sterilization methods. Bioflex A80 MB is only suitable for low-temperature disinfection–sterilization, as high-temperature treatments cause surface cracking. Neither resin is appropriate for cryogenic conditions due to the risk of brittleness. Further research into post-sterilization properties is essential to ensure the safety and clinical reliability of these materials in cardiac procedures.

## 1. Introduction

The integration of computer-aided design (CAD) and additive manufacturing in the medical field has enabled rapid in-house prototyping and production of 3D-printed, customized models [1,2]. Our group previously introduced the first 3D-printed surgical guides for cardiac arrhythmia procedures by modeling target ablation sites for patients with Brugada Syndrome and ischemic ventricular tachycardia [3,4]. They address a critical limitation in hybrid ablation procedures, where mapping challenges can lead to incomplete ablation and post-operative arrhythmia recurrence [5].

The CAD models we previously designed to address this unmet surgical need were created using advanced imaging and mapping data, then printed into electro-anatomically accurate, tactile guides [3,4]. They are intended for temporary application on the heart during ablation, providing the surgeon with a clear, predefined view of arrhythmogenic areas and precise target sites for ablation [3,4].

Resin-based 3D printing materials are commonly used to produce surgical guides, but a significant number are not validated to meet hospital standards or comply with Medical Device Regulations. To be deemed eligible for sterile surgical guide applications near ablative (radiofrequency or cryo-) energy, the material must withstand thermal, mechanical, and chemical stresses, remain biocompatible, and maintain its structural integrity after disinfection and sterilization at temperatures up to 134 °C. Ideal materials should also be non-conductive, dimensionally stable, and flexible enough to conform to the beating heart in minimally invasive surgery.

Effective disinfection and sterilization are critical for clinical safety but can alter the chemical and physical structure of 3D-printed resins, potentially compromising performance [6,7,8]. Pre-sterilization cleaning and disinfection are essential to reduce microbial load, achieved through enzymatic detergents followed by disinfection with EPA-approved agents [9]. High-level microbial decontamination employs physical methods (steam, dry heat), chemical agents (ethylene oxide, hydrogen peroxide), or radiation (ionizing, UV), applied through high- or low-temperature sterilization processes [9,10]. Autoclaving (steam sterilization) is the most common and accessible method for sterilizing 3D-printed components at 121 °C, 132 °C, or 134 °C [10,11,12]. It is cost-effective, chemical-free, and residue-free, but may cause breakage of physical crosslinks and secondary crosslinking during cool-down, especially in heat-sensitive thermoplastics with transition temperatures near autoclave conditions [13]. Vaporized hydrogen peroxide (VHP) sterilization offers an alternative for heat-sensitive polymers, generating reactive species that disrupt microbial cells, but it carries a risk of sterilant residue [14].

Since sterilization can degrade materials by oxidation, structural changes, and residue accumulation, it is essential to test key properties such as glass transition temperature (T_g_) and mechanical stability to ensure clinical safety.

MED625FLX™ and Bioflex A80 MB™ are flexible, medical-grade 3D printing resins with potential for use as surgical guides due to their soft surface and mechanical properties that balance rigidity and pliability. MED625FLX, used in dental applications, offers moderate flexibility and a silicone feel that mimics soft tissue, making it ideal for epicardial contact and minimizing trauma. It has been proven to support 24 h of mucosal contact, with defined post-processing and approved sterilization under high steam temperatures [15]. Bioflex A80 MB provides higher elasticity and resolution, low shrinkage, and, according to the manufacturer, is ultra-pure, safe, and non-cytotoxic after post-curing and cleansing.

Although the datasheet for MED625FLX suggests steam sterilization (132 °C, 4 min) and gamma sterilization (25–50 kGy), issues such as deformation and altered coloring may occur. Key data on post-sterilization material properties and biocompatibility are unspecified in both materials, further underscoring the need for this research.

With the rise in in-house 3D printing, the production and quality of surgical guides have shifted from external service providers to clinicians, increasing the importance of meeting regulatory standards and validated sterilization protocols [16,17,18].

Thus, herein, we investigate how disinfection and sterilization affect the thermal and mechanical properties of MED625FLX™ and Bioflex A80 MB™. This study addresses a critical gap in identifying durable, sterilizable 3D-printed materials for surgical guides in ablation procedures. The null hypothesis states that neither disinfection nor H_2_O_2_ nor steam sterilization significantly alters these properties. 

## 2. Materials and Methods

**Figure 1:**
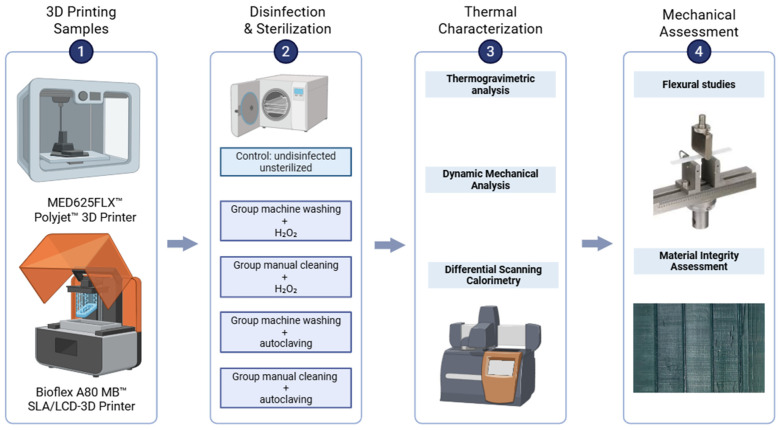
Schematic diagram of the study. Created in https://BioRender.com.

A schematic overview of the methodology is shown in Figure 1.

### 2.1. Materials and 3D Printing Process

#### 2.1.1. MED625FLX

MED625FLX™, CL (Stratasys, Minneapolis, MN, USA) is a clear, thermoplastic dental resin used with polymer jetting (PolyJet) technology [19]. It is a non-toxic, medically certified biocompatible (ISO 10993-1 [20]) (this certification applies to non-sterilized samples). MED625FLX was approved for prolonged skin contact (over 30 days), short-term contact with mucosal membranes (up to 24 h), and limited contact with tissue and bone. MED625FLX has a Shore hardness of 73–77 A, elongation at break of 45–55%, tensile strength of 3.0–5.0 MPa, and tensile tear resistance of 8–12 Kg/cm [19]. Table S1 (Supplemental Material) lists material properties. The full chemical composition is proprietary.

3D Printing Process

MED625FLX is printed with PolyJet technology, a material jetting technique where liquid photopolymers are jetted and cured layer by layer using UV light [19,21]. Specimens were produced using a PolyJet™ 3D printer (Objet260 Connex 1, Stratasys, Minneapolis, MN, USA), operating in high-speed, high-quality mode with a 28 µm layer thickness. This single material with glossy printing enhances layer resistance.

Post-processing

Non-biocompatible SUP705™ support material (Stratasys, Minneapolis, MN, USA) was removed by rinsing with a waterjet before and after soaking in 1% sodium hydroxide solution (3 h at room temperature), followed by a 30 min soak in 99% isopropanol and air-drying.

#### 2.1.2. BIOFLEX A80 MB

Bioflex A80 Monomer Based™ (Bioflex) (3Dresyns, Barcelona, Spain) is a non-toxic, biocompatible photopolymer resin (this certification applies to non-sterilized samples) with a Shore hardness of A80, elongation under 100%, Young’s modulus under 90.0 MPa, tensile and flexural strengths less than 15.0 MPa (3Dresyns.com). For this study, we optimized Bioflex for multilayer liquid crystal display (LCD) printers and enhanced it with green additives and fine-tuner FT2P for improved resolution. Table S1 lists known material properties. The chemical composition is not provided by the manufacturer.

3D Printing Process

We designed the specimen meshes using Blender 4.1 (dimensions further detailed below) and exported them as a suitable file format (*.stl*) for 3D printing. The printer and resin containers were cleaned, and printing took place in an enclosed, air-filtered chamber to minimize contamination. Settings were calibrated for our Anycubic Photon Mono X SLA/LCD-3D printer (Anycubic, Shenzhen, China) to ensure reliable and cleanable specimens. This technology cures liquid photopolymer resin layer by layer with UV light [21]; each layer is projected onto an LCD screen and cured for 15 s as the build platform descends, gradually solidifying the uncured material into an elastomer.

Post-processing

After printing, Bioflex models were cleaned in an ultrasonic bath with biocompatible resin cleaner (SKU: P20397, www.3Dresyns.com) at 40–45 °C for 20 min, then detached from support structures. A second ultrasonic wash (40–45 °C; 30 min) removed uncured resin. Specimens were UV-cured (four 15 min exposures at varying angles), dipped in a bio-safe detoxifying solution (SKU: P11197, www.3Dresyns.com) for washing and cleaning 3D prints (5 min), rinsed with deionized water, and heated to 70–80 °C in biocompatible resin cleaner (SKU: P20397, www.3Dresyns.com) to eliminate leachable toxins.

### 2.2. Disinfection and Sterilization Methods

We divided post-processed samples into four groups based on disinfection and sterilization methods (Table 1):

Low-Temperature Washing

Manual cleaning was performed using Aniosyme XL3 solution (Laboratoires Anios, Lezennes, France) at a final working concentration of 0.5% (*V*/*V*) (25 mL in 5 L water) with a soaking time of 5 min, followed by chemical disinfection using 70% (*V*/*V*) ethanol. Each step was carried out once. Samples were air-dried and placed on metal supports before sterilization.

High-Temperature Washing

The specimens were cleaned in an automated cleaning system (Miele, Princeton, NJ, USA) with a 1.5 h cycle, including a pre-rinse alkaline washing (Mediclean Forte, Dr. Weigert, Hamburg, Germany) and an alkaline thermal disinfection (Septoclean, Dr. Weigert, Hamburg Germany) at approximately 95 °C, followed by a drying phase.

H_2_O_2_ Sterilization

Vaporized hydrogen peroxide (VHP) sterilization was performed at 60 °C using the Sterrad© Sterilization System (Johnson & Johnson, New Brunswick, NJ, USA) for ~60 min.

Autoclaving

Steam sterilization was performed at 134 °C for 4 min using autoclaving with fractionated pre-vacuum (cycle of ~60 min) between 3045 and 3101 mbar, followed by cooling and a 12 h degasification period.

Sterilized samples were individually packaged with sterile barrier systems per ISO 11607-1:2019 [22]. All processes were performed at the University Hospital of Brussels’ Central Sterilization Unit.

### 2.3. Thermal Characterization

Thermogravimetric Analysis (TGA) was performed to assess thermal stability and decomposition behavior in MED625FLX and Bioflex. TGA thermograms were recorded on a TA Instruments Q5000 (TA Instruments, New Castle, DE, USA) under air atmosphere (with a flow rate of 25 mL·min^−1^). A heating rate of 10 K min^−1^ was applied up to a temperature of 600 °C on samples weighing 2–3 mg. Three samples were measured per group.

Differential scanning calorimetry (DSC) was performed to evaluate the thermal transitions and the glass transition temperature (T_g_) of both materials. DSC experiments were performed using a Discovery DSC 250 (TA Instruments, New Castle, DE, USA) with a refrigerated cooling system. All experiments were performed in Tzero aluminum pans in hermetic conditions using nitrogen as purge gas. A heating and cooling rate of 10 K·min^−1^ was employed between −50 °C and 90 °C on samples weighing 5–8 mg. Three samples were measured per group.

Dynamic mechanical analysis (DMA) was conducted to assess the viscoelastic behavior of MED625FLX and Bioflex by measuring changes in the storage modulus (E′), loss modulus (E″), and loss factor (tan δ) as a function of temperature. DMA tests were performed on DMA Q800 (TA Instruments, New Castle, DE, USA), equipped with a gas cooling accessory that allows cooling below −100 °C. Dynamic measurements on rectangular samples (24.0 mm length, 5.0 mm width, 1.00 mm thickness) were performed in tension mode using a film tension clamp. A temperature ramp was applied with a heating and cooling rate of 3 K.min^−1^ in the range of −80 °C to 80 °C with a constant frequency of 1 Hz and oscillatory strain of 0.1%. Three samples were tested for each group. All samples were handled and clamped in the same way to avoid introducing variation. Tan δ, the ratio of E″ to E′, was recorded, and T_g_ was determined from its peak temperature.

### 2.4. Flexural Testing

Flexural tests were conducted to evaluate flexural properties, including the impact of disinfection–sterilization. We used a three-point bending setup on a Universal Testing Machine (Model 5885H, Instron, PA, USA) at room temperature (Figure S1; Supplementary Material). A total of 50 specimens (127 × 12.7 × 3.2 mm; n = 25/material) were printed as per ASTM 790-17 [23] standards and tested under vertical loading at a strain rate of 0.10 mm/mm/min and a crosshead motion rate of 13.7 mm/min until failure (500 N load cell; support span 51.2 mm) [24]. We measured the maximum load at the highest displacement allowed by the setup, with no fractures observed during bending tests. Larger deflections could be achieved if the setup permitted. Additionally, as a trial for assessment of differences in behavior under greater deformations, larger MED625FLX specimens (MED625FLX-L) (400 × 25 × 6.5 mm; n = 15) were tested with a crosshead motion rate of 27.73 mm/min and a 104 mm support span. We calculated the flexural modulus (*E_f_*) using the formula

$$E_{f}=\frac{L^{3}m}{4bd^{3}}$$

where *L* is the support span (mm), *m* is the slope of the load–deflection curve (N/mm), *b* is specimen width (mm), and *d* is specimen depth (mm). We derived the stress–strain curves from the force–displacement data and calculated the elastic modulus by measuring the slope within the range of 20–60% of the peak force. The R^2^ value was used to validate the linearity of the curve. A high R^2^ value, close to 1, confirmed a strong force–displacement relation, allowing us to confidently calculate the slope, and thus, elastic modulus, in the linear part of the stress–strain curve.

### 2.5. Material Integrity Assessment

We performed a thorough visual inspection on all post-processed MED625FLX and Bioflex specimens before and after disinfection–sterilization to identify macroscopic deformities such as crazing, cracking, breakage, loose fragments, or color alterations. Specimens that showed cracking, For specimens that exhibited cracking, the depth of the cracks was assessed using micro-computed tomography (CT) (TESCAN UniTOM XL, Brno, Czech Republic) with a voxel size of 7.5 µm and source resolution of 3 µm.

### 2.6. Statistical Analysis

Experimental data are expressed as mean ± standard deviation (SD). The elastic modulus was analyzed using one-way ANOVA, followed by Bonferroni tests (α = 0.05), with *p*-values < 0.05 deemed significant. All analyses were performed using SPSS v.26 (IBM, Armonk, NY, USA). A post hoc power analysis was performed using G*Power 3.1.

## 3. Results

### 3.1. Material Integrity

**Figure 2:**
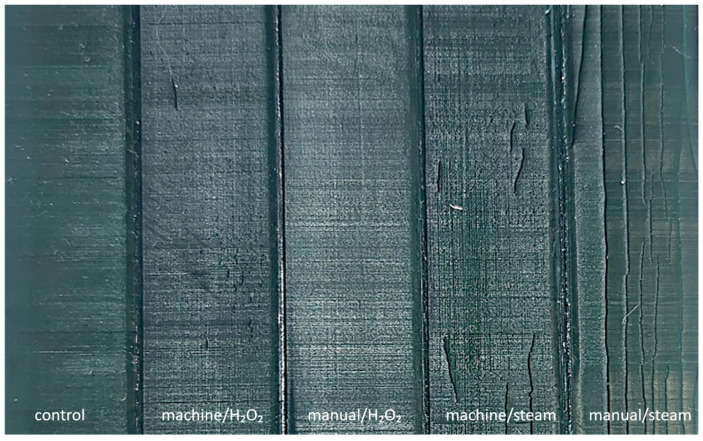
Macroscopic overview of sterilized Bioflex Prints. All groups except manual/H_2_O_2_ demonstrate consistent superficial cracking in various patterns, with group manual/steam showing the most prominent surface cracking. Crack dimensions observed across groups are as follows: Machine/H_2_O_2_—lengths 1.0–10.0 mm, depths 0.17–0.20 mm; Machine/Steam—lengths 2.0–30.0 mm, depths 0.420–0.455 mm; Manual/Steam—lengths 3.0 mm to full-length fractures, depths 0.576–0.655 mm.

Visual inspection of post-processed, sterilized MED625FLX specimens showed no macroscopic deformities, crazes, cracks, color alteration, breakage, or loose fragments. Bioflex samples from all groups except manual/H_2_O_2_ demonstrated superficial cracking in various patterns, without complete fractures or loose fragments (Figure 2). Micro-CT analysis was performed on these specimens to assess crack depth. Group machine/H_2_O_2_ exhibited diffuse, short vertical, superficial cracks, ranging from 1.0 mm to 10.0 mm in length, and crack depth ranging from 0.17 mm to 0.20 mm. Manual/H_2_O_2_ samples remained fully intact. Machine/steam samples showed more pronounced cracks, similar in pattern to the machine/H_2_O_2_ group, with crack lengths ranging from 2.0 to 30.0 mm, and depths between 0.420 mm and 0.455 mm (Figure S2; Supplemental Materials). Manual/steam samples exhibited extensive longitudinal cracks, ranging from 3.0 mm in length to full-length fractures, and depths between 0.576 mm and 0.655 mm (Figure S2). Additionally, subtle bulging of the material was observed in this group on micro CT imaging. Subtle color fading was noted in the manual/H_2_O_2_ Bioflex group. No crazing was observed.

### 3.2. Flexural Modulus

Both MED625FLX and Bioflex exhibit high bending flexibility with no fracturing during the experiments, followed by complete elastic rebound without permanent deformation or cracking. Figure S3 plots the bending load–displacement curves for MED625FLX and Bioflex under bending conditions until the maximum applied load. MED625FLX showed steeper curves compared to Bioflex, indicating higher rigidity. As aforementioned, setup constraints need to be considered, as the test could continue to larger deflections if the setup allowed. The elastic modulus was analyzed using one-way ANOVA and post hoc tests with Bonferroni (α = 0.05), with *p*-values < 0.05 and Bonferroni-corrected *p*_a_-values < 0.005 deemed significant. A post hoc power analysis yielded power values of 0.90 for MED625FLX-S, 0.54 for MED625FLX-L, and 1.00 for Bioflex, indicating adequate power for MED625FLX-S and Bioflex, but low power for MED625FLX-L.

**Figure 3:**
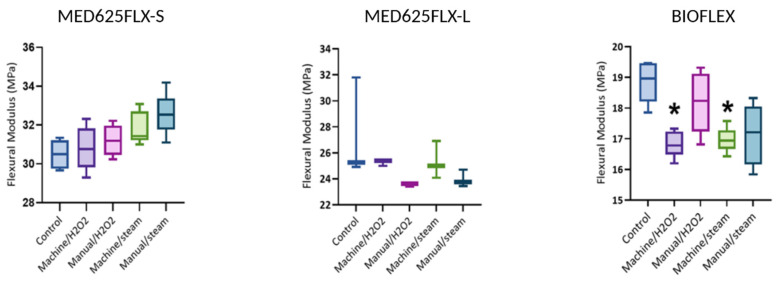
Boxplots of flexural moduli for different sterilized groups and materials compared to the control. MED625FLX-S: Flexural modulus showed minor, non-significant increases across sterilization groups. MED625FLX-L: Remained relatively stable with minor, non-significant variations. Bioflex showed a significant decrease in groups machine/H_2_O_2_ and machine/steam when compared to the control (shown by *).

Table S2 in the Supplemental Material provides a complete summary of all flexural moduli and statistical analysis. Boxplots of flexural moduli are shown in Figure 3.

In group machine/H_2_O_2_, the flexural modulus of MED625FLX-S showed a slight non-significant increase compared to its control group, while MED625FLX-L exhibited a slight decrease. Bioflex, however, showed a significant decrease in modulus (16.85 ± 0.43 MPa, *p* < 0.001) compared to its control. In group manual/H_2_O_2_, the modulus of MED625FLX-S increased slightly compared to unsterilized samples, while those of MED625FLX-L and Bioflex decreased, with Bioflex exhibiting the highest variability. None of these changes proved significant. For group machine/steam, the flexural modulus of MED625FLX-S showed a slight increase compared to its control, whereas MED625FLX-L presented a slight decrease compared to its control, both without significant differences. Bioflex’s modulus, however, decreased significantly (16.96 ± 0.41 MPa, *p* = 0.001). In the group manual/steam, MED625FLX-S showed the highest flexural modulus in this group, while MED625FLX-L exhibited a lower modulus compared to its control. Bioflex’s modulus showed high variability and decreased compared to unsterilized Bioflex samples, though not significantly.

### 3.3. Thermal Characterization

#### 3.3.1. Thermogravimetric Analysis

**Figure 4:**
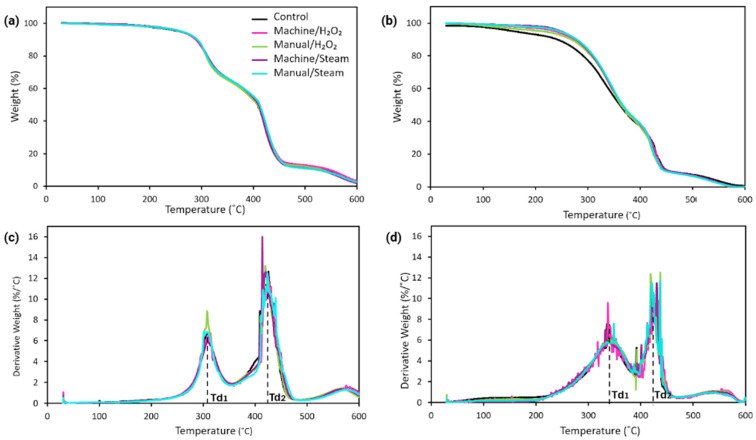
Thermogravimetric analysis curves for (**a**) MED625FLX and (**b**) Bioflex; and the derivative thermogravimetric (DTG) curves for (**c**) MED625FLX and (**d**) Bioflex sample sets. Td_1_: Average mass loss temperature for the first step, taken as the maximum point of the first peak from the DTG curve. Td_2_: Average decomposition temperature for the second step, taken as the maximum point of the second peak from the DTG curve.

Thermal stability and decomposition behavior were studied by TGA under an air atmosphere. Figure 4 shows the TGA and derivative thermogravimetric (DTG) curves for MED625FLX and Bioflex samples for each group. To gain further insight into the thermal properties, mass loss in percentage at 100 °C, as well as three characteristic temperatures, were summarized in Table 2.

TGA thermograms of the MED625FLX and Bioflex samples (Figure 4a,b) both show a two-step weight loss under an air atmosphere. For unsterilized MED625FLX samples, the main mass loss temperatures are shown as 309 ± 1 °C (Td_1_) and in the second step as 423 ± 2 °C (Td_2_) (Figure 4c). For the unsterilized Bioflex samples, the main decomposition temperatures are 326 ± 6 °C (Td_1_) and 425 ± 3 °C (Td_2_) (Figure 4d). Comparing the first mass loss temperatures of unsterilized samples, Bioflex exhibited 17 °C higher thermal stability than MED625FLX. However, the second-step decomposition temperatures were similar for both materials.

For MED625FLX samples, the sterilized groups showed slightly higher Td_5_ values compared to the control samples. Groups machine/H_2_O_2_, manual/H_2_O_2_, machine/steam, manual/steam exhibited Td_5_ values of 258 ± 2 °C, 259 ± 7 °C, 256 ± 3 °C, and 258 ± 2 °C, respectively, while the control samples had a Td_5_ value of 247 ± 8 °C, suggesting that sterilization slightly increased Td_5_. However, it did not affect the primary mass loss temperatures, Td_1_ and Td_2_, of the polymer. Additionally, no significant differences were observed among the sterilized groups; thus, the sterilization method had no impact on the decomposition behavior of the MED625FLX polymers.

For Bioflex samples, the decomposition temperature at 5% weight loss for the unsterilized sample was found as 163 ± 7 °C, which was increased after sterilization up to 240 ± 5 °C, 221 ± 6 °C, 252 ± 4 °C, and 244 ± 2 °C for groups machine/H_2_O_2_, manual/H_2_O_2_, machine/steam, manual/steam, respectively, indicating that the increase in Td_5_ is likely due to the removal of volatile or low molecular weight components during sterilization. Group machine/steam showed the highest Td_5_, indicating that high-temperature washing and steam sterilization effectively removed volatiles below the first decomposition temperature better than other methods in Bioflex.

Mass loss percentages at 100 °C revealed that for MED, group machine/H_2_O_2_ (0.66%) is similar to the control group (0.67%). Group manual/H_2_O_2_ and manual/steam show slightly lower mass loss (0.53%), while group machine/steam exhibits a higher value (0.80%). For Bioflex, the control group shows the lowest mass loss (0.42%). Group machine/H_2_O_2_ shows the highest mass loss (1.04%), while group manual/H_2_O_2_ and group machine/steam exhibit moderate values (0.63% and 0.9%, respectively). For all sample sets, the final residue at temperatures above 500 °C for both sets was minimal (approximately 2–5%), suggesting near-complete decomposition in an air atmosphere.

#### 3.3.2. Differential Scanning Calorimetry

**Figure 5:**
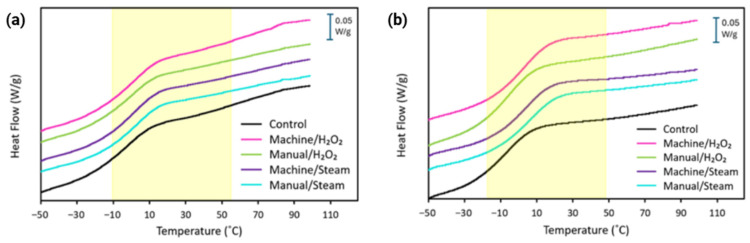
DSC thermograms (exo down) of (**a**) MED625FLX and (**b**) Bioflex groups, revealing a broad Tg in the highlighted area.

Figure 5a shows the first heating curve from DSC analysis of MED625FLX, and Figure 5b for Bioflex samples. The DSC thermograms show a broad T_g_ transition for each sample set, as shown in the highlighted area on the curves (Figure 5).

Tg values obtained from DSC are summarized in Table 3. Average values and standard deviations were calculated from three independent measurements for each sample. For MED625FLX samples, the control group shows an average Tg of −4 ± 2 °C, while Bioflex control samples showed a lower Tg of −11 ± 1 °C. Among the sterilized MED625FLX groups, Tg was calculated as −1 ± 1 °C, −2 ± 1 °C, 1 ± 1 °C, and 1 ± 2 °C for groups machine/H_2_O_2_, manual/H_2_O_2_, machine/steam, manual/steam, respectively. No significant change was observed between different sterilization methods, nor compared to the unsterilized control samples for MED625FLX samples. On the other hand, sterilization appeared to cause an increase in the Tg values for Bioflex samples, with a maximum shift of 11 °C in manual/steam, resulting in a Tg of 0 ± 2 °C. Group machine/H_2_O_2_ showed a slightly lower Tg of −9 ± 1 °C compared to the other sterilized groups. For both MED625FLX and Bioflex, no additional thermal transitions, such as melting or crystallization, were observed up to 90 °C in either material, showing the amorphous nature of the material.

#### 3.3.3. Viscoelastic Behavior

**Figure 6:**
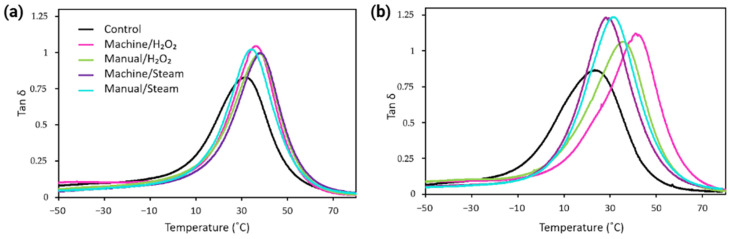
DMA measurement of tan δ for (**a**) MED625FLX and (**b**) Bioflex sample sets, showing the transition from a glassy state (low tan δ), through the glass transition temperature (T_g_, peak tan δ), to a rubbery plateau at higher temperatures.

DMA data was analyzed using TA Universal Analysis software (v5.7, TA Instruments, New Castle, DE, USA). Figure 6 shows the loss factor (tan δ) results for both materials. Figure S4 (Supplemental Materials) depicts exemplary DMA curves from one sample per group. T_g_ was identified as the temperature where tan δ reached its peak, indicating a significant increase in polymer chain mobility. The DMA thermograms exhibited broad tan δ peaks, which are consistent with the T_g_ transitions observed in DSC results.

The T_g_ values measured by DMA (Table 3) were consistently higher than those obtained by DSC for both materials. For MED625FLX samples, the control group shows a T_g_ of approximately 32 ± 1 °C. The sterilized groups machine/H_2_O_2_, manual/H_2_O_2_, machine/steam, and manual/steam showed average T_g_ values of 36 ± 1 °C, 36 ± 2 °C, 36 ± 1 °C, and 35 ± 1 °C, respectively. These values indicated no significant variation between sterilized groups, and there was no notable shift in T_g_ due to the sterilization process when compared to control samples, consistent with DSC results.

In contrast, Bioflex samples demonstrated a T_g_ of 22 ± 1 °C in the control group, while sterilization led to an increase in T_g_, with values up to 38 ± 3 °C for machine/H_2_O_2_ samples. We observed a similar increase in T_g_ in the other sterilized groups, showing T_g_ values of 35 ± 1 °C, 35 ± 1 °C, and 31 ± 2 °C for groups manual/H_2_O_2_, machine/steam, and manual/steam, respectively, demonstrating the impact of sterilization. Group machine/H_2_O_2_ showed the largest shift in T_g_ by 16 °C.

## 4. Discussion

This study addresses a critical challenge in the clinical application of 3D-printed materials: the impact of sterilization on material properties—a vital aspect still underexplored in the literature and manufacturer data. We conducted the first study evaluating MED625FLX and Bioflex 3D printing resins for use in cardiac ablation surgical guides, focusing on how disinfection and sterilization affect their thermal and mechanical properties. While previous studies have addressed the sterilization of surgical guides, our investigation focuses on how different disinfection methods additionally impact these materials.

### 4.1. Mechanical Integrity

MED625FLX samples showed no surface defects after sterilization. In contrast, Bioflex exhibited superficial cracks in all high-temperature groups, indicating its sensitivity to thermal stress. Autoclaving likely accelerated hydrolysis through heat and moisture, leading to swelling and degradation. Shuen et al. reported cracking in 9 of 15 methacrylate-based resin surgical guides after steam sterilization at 135 °C for 4 min, and in 7 of 15 guides after autoclaving at 121 °C for 30 min [25]. Fuentes et al. found that steam sterilization caused greater degradation of polymer matrices than dry heat in PLA, PETG, and CPE due to combined thermal and hydrolytic effects, with PLA samples exhibiting brittle fractures [12]. Though the tested materials differ, the process is similar. Further on, the manual/steam group exhibited the deepest and most extensive cracks, indicative of environmental stress failure. Combined chemical (ethanol, H_2_O_2_), thermal, and mechanical stress likely accelerated hydrolysis and material degradation. The manual/H_2_O_2_ group showed no cracking, but demonstrated reduced stiffness, suggesting low-temperature H_2_O_2_ caused mild oxidative stress and subtle changes in crosslinking. Popescu et al. studied whether the combination of disinfection–sterilization could degrade 3D printed acrylonitrile butadiene styrene parts. They found that parts treated with acetone vapors and sterilized with VHP showed reduced mechanical performance compared to untreated controls, but no material degradation resulting from disinfectants was observed [26]. Moreover, Bosc et al. found H_2_O_2_ had minimal impact on acrylonitrile butadiene styrene guide morphology and surface roughness [27].

### 4.2. Flexural Modulus

Three-point bending tests demonstrated ductility in both materials, confirming their ability to withstand deformation under the tested stress conditions. MED625FLX maintained structural integrity under deformation in all sterilization groups, even in larger specimens. In MED625FLX, autoclaving consistently increased stiffness, though insignificantly. This can be potentially attributed to thermal effects like crosslinking and densification. Post hoc power analysis confirmed low statistical power for MED625FLX-L, as expected given the small sample size (n = 3/group). Thus, its results should be interpreted with caution. The only significant impact during flexibility studies was observed on Bioflex when stiffness decreased when subjected to high-temperature mechanical washing and H_2_O_2_/steam treatment. As both groups were exposed to high-temperature machine disinfection, this is likely due to hydrolysis, which weakens the polymer chains. Consistent with these results, manual/steam also resulted in a reduction in stiffness, albeit not statistically significant.

Few publications in the past decade have examined the effect of post-curing disinfection and sterilization of surgical guides. Similarly to our results, Eveland et al. found that H_2_O_2_ caused no significant changes in the flexural mechanical properties, maintaining material integrity of 3D printed surgical guides (Stratasys’ MED610™, MED615™, and MED620™; Formlabs’ BioMed Clear™ and BioMed Amber™) [28]. Van Dal et al. found that the flexural strength of Formlabs BioMed Clear™ did not exhibit significant changes following H_2_O_2_ exposure, but it did increase after autoclaving [22]. Kirschner et al. showed that autoclaving caused a slight reduction in the flexural modulus of 3D-printed resins for surgical guides when exposed to 134 °C (2 bar, 5.5 min) [29]. However, no significant changes were observed at 121 °C (1 bar, 20.5 min). A pilot study by Török et al. found that disinfection did not impact the mechanical properties of 3D-printed surgical templates made from Objet MED610™ (Stratasys) and that steam sterilization at 121 °C and plasma sterilization caused no significant dimensional or material changes in the tested drill templates [30]. Furthermore, Zaborniak et al. proved that steam sterilization in MED610™ produced smoother surfaces but also caused a slight decrease in bending strength, potentially restricting its suitability for load-bearing temporary implants [31]. Rynio et al. found that steam sterilization at 121 °C did not deform resin-based models and that VHP sterilization was safe for both flexible and rigid resins [10]. Similarly, Marei et al. demonstrated that steam sterilization had minimal impact on the dimensional changes in surgical guides [32]. Contrarily, Pop et al. found that autoclaving (121 °C; 134 °C) modified mechanical properties and induced brittle behavior in SLA- and digital light processed-printed surgical guides, making them unsuitable choices for guide use [33]. Labakoum et al. demonstrated that high-temperature sterilization alters the mechanical and geometric properties of 3D-printed surgical guides made with LCD technology [8]. Similarly, Lan et al. reported that autoclaving led to notable deformation, decreased accuracy, and increased 3D deviations in photopolymerized resin surgical templates [34]. Shaheen et al. found significant morphological changes in autoclaved orthognathic splints, reducing their reliability after heat sterilization [35].

Although both materials pass flexural tests, they are not ideal for folding and insertion through a 12 mm trocar in minimally invasive thoracoscopic surgery. Future work will explore more flexible resins or alternative materials, such as silicones.

### 4.3. Decomposition Behavior

Bioflex demonstrated better thermal stability than MED625FLX at higher temperatures, particularly in group manual/H_2_O_2_ (Td_2_ = 432 °C). The initial mass loss temperatures observed are likely attributable to the decomposition of additives, with continuous mass loss before Td_1_ suggesting gradual additives removal; consistent with the observed increase in Td_5_ after sterilization. Td_2_ likely represents degradation of the polymer backbone. Moreover, minimal mass loss is observed at 100 °C, and can be attributed to water evaporation. These results confirm that both materials remain thermally stable within the clinical working temperature range, even under extreme conditions of unintended contact with the radiofrequency ablation instrument. Moreover, unsterilized Bioflex samples showed the best resistance to early decomposition (lowest weight loss at 100 °C) compared to all other groups. However, sterilized samples (specifically machine/steam in MED625FLX; machine/H_2_O_2_ and manual/steam in Bioflex) showed greater mass loss at 100 °C.

Sterilization increased Td_5_ for all sterilized groups compared to the control. Bioflex group machine/steam was the most thermally stable polymer compared to other groups, suggesting washing machine cleaning, followed by steam sterilization, most effectively reduced volatiles or low molecular weight components below the first main mass loss temperature. Similar trends were observed for MED625FLX, indicating that disinfection–sterilization eliminates low molecular weight fragments or impurities that typically degrade at lower temperatures. However, no significant differences were observed between the sterilized and unsterilized samples regarding the maximum decomposition temperatures, indicating that the sterilization methods did not markedly affect general mass loss.

However, as the chemical formulations of both resins are proprietary, the exact mechanisms behind post-disinfection/sterilization property changes remain unclear. This also limits the ability to tailor sterilization treatments compatible with the resin. To the best of our knowledge, no other studies have examined the thermal decomposition of photopolymer resins after disinfection–sterilization.

### 4.4. Glass Transition Temperature

DSC and DMA analyses confirm that the materials exhibit amorphous elastomeric behavior, retaining their flexibility and softness while maintaining performance at physiological temperature and during unintended instrumental contact with the border of the guide. However, under cryo-energy conditions at lower temperatures, they become glassy and brittle, making them unsuitable for cryo-applications.

DMA results showed that for MED625FLX samples, there was no significant change in T_g_ between the control and sterilized groups, consistent with DSC findings. Contrarily, Bioflex showed a significant T_g_ increase after sterilization, with the largest shift of 16 °C in machine/H_2_O_2_ samples. This could be attributed to high-temperature disinfection combined with H_2_O_2_, either by promoting additional network formation or by removal of additives and low molecular weight components. Smaller but similar T_g_ increases were observed in all other groups. Bioflex is a monomer-based photopolymer resin; thus, additional heat exposure during disinfection and sterilization can further enhance monomer conversion and crosslinking, resulting in altered properties and an increased T_g_. However, the minimal T_g_ differences between sterilized groups suggest no additional network reactions and are likely clinically insignificant.

Thus, stability changes are likely only due to the removal of low molecular weight compounds, such as unreacted materials and additives, aligning with TGA findings. The discrepancy between DMA and DSC results may stem from the different properties measured by each technique. A compounding effect from thermal lag could also contribute to an overestimation of temperature in DMA. Advanced techniques like Fourier Transform Infrared Spectroscopy and Mass Spectrometry should be conducted to further explore chemical changes during thermal degradation.

These thermal characterization results demonstrate that both materials exhibit favorable thermal stability and flexibility with a higher temperature range under simulated clinical radiofrequency ablation conditions, with minimal mass loss (≤1.04%) up to 100 °C. However, novel materials with lower T_g_ values should be considered for cryo-applications, such as silicone rubbers (T_g_~−115 °C to −120 °C), which remain flexible at cryogenic temperatures.

Thus, taken together, the null hypothesis is accepted for MED625FLX, as sterilization and disinfection did not significantly affect its mechanical or thermal properties. For Bioflex, however, the null hypothesis is rejected due to changes in material integrity, a significant reduction in flexural modulus, and minor shifts in thermal behavior.

Although the low-temperature method seemed more suitable for Bioflex, H_2_O_2_ has notable limitations, as it may leave sterilant residues. Polymers are also prone to oxidative damage, as hydroxyl free radicals can cause crosslinking [14]. Additionally, H_2_O_2_ has poor penetration in complex surgical guides due to suboptimal penetration and insufficient vapor dispersion, limiting its effectiveness for designs with suction components [7,13]. While it better preserves material integrity than high-temperature methods, H_2_O_2_ is most suitable for simpler, non-absorbent polymer surfaces [13].

### 4.5. Additional Considerations

A crucial consideration is how post-printing processes affect polymer leachability and cytotoxicity [36,37,38,39,40]. Under the European Medical Device Regulation, devices intended specifically for use in direct contact with the heart are classified as Class III, the highest risk category (Regulation (EU) 2017/745, eur-lex.europa.eu), requiring stringent sterilization and safety standards. While ISO-certified biocompatibility of 3D-printing materials provides a baseline, the chemical and thermal treatments used in post-processing, disinfection, and sterilization can significantly impact their final biological safety [28,37]. Moreover, polymeric devices, particularly in-house 3D-printed surgical guides, pose unique challenges as they can absorb solvents and retain residues, which may introduce contaminants [13].

### 4.6. Limitations and Future Considerations

The main limitations of this study are the small sample size and the restriction to two 3D printing materials. Future studies should use larger data sets and include a broader range of materials (e.g., flexible resins, silicones). Additionally, while we selected autoclaving and VHP due to their common use and availability at our center, future studies should also examine other sterilization modalities—dry heat, steam at 121 °C, gamma irradiation, UV—and their potential effects. Further on, the chemical composition of the resins used in this study is not fully disclosed by the manufacturers, which limits the interpretation of underlying mechanisms related to the disinfection–sterilization process. Moreover, this limitation hinders benchmarking against other materials and comparability with other studies. Additionally, flexural testing was prioritized due to its clinical relevance; however, future work should also include tensile testing, as well as post-sterilization dimensional accuracy and folding tests mimicking minimally invasive procedures. Further research is needed on the impact of sterilization on the in vitro cytotoxicity of 3D-printed resins. Additionally, toxicological assessments and chemical residue analyses are essential to establish acceptable leachable limits, which should be addressed in future studies. Finally, preclinical in vivo studies are needed to assess material performance under surgical conditions, including exposure to radiofrequency and cryoablation.

## 5. Conclusions

From this preliminary study, the following conclusions can be drawn: (i) both high and low-temperature disinfection, as well as steam and H_2_O_2_ sterilization, effectively preserve the integrity and flexibility of MED625FLX guides; (ii) low-temperature disinfection and H_2_O_2_ sterilization preserve material integrity in Bioflex; (iii) high-temperature disinfection and sterilization should be avoided in Bioflex due to surface cracking; (iv) neither resin is suitable for cryogenic conditions due to the risk of brittleness in lower temperature applications. Further research into sterilization protocols is essential to ensure the reliability of these resins as surgical guides in cardiac ablation procedures.

## Figures and Tables

**Table 1:** Summary of disinfection and sterilization treatments used in this study.

Group	Cleaning and Disinfection Method	Sterilization Method with T °C and Cycle Duration
Control	None	None
Machine/H_2_O_2_	Washing machine cleaning with Mediclean Forte, thermal disinfection (~95 °C). Cleaning cycle: 1.5 h	VHP: H_2_O_2_ gas plasma at 60 °C (Sterrad System)~60 min
Manual/H_2_O_2_	Manual cleaning (Aniosyme XL3, 0.5% (*V*/*V*), 25 mL in 5 L water) + 70%(*V*/*V*) ethanol ~5 min. Single cycle.	VHP: H_2_O_2_ gas plasma at 60 °C (Sterrad System)~60 min
Machine/steam	Washing machine cleaning with Mediclean Forte, thermal disinfection (~95 °C). Cleaning cycle: 1.5 h	Autoclave at 134 °C, 4 min plateau, fractionated pre-vacuum~60 min
Manual/steam	Manual cleaning (Aniosyme XL3, 0.5% (*V*/*V*), 25 mL in 5 L water) + 70% (*V*/*V*) ethanol ~5 min. Single cycle.	Autoclave at 134 °C, 4 min plateau, fractionated pre-vacuum~60 min

**Table 2:** TGA data of (un)sterilized MED625FLX and Bioflex sample groups. Td_5_: Temperature at which 5 percent weight loss was observed. Average values and standard deviations were calculated from three independent measurements for each sample. Td_1_: Average mass loss temperature for the first step. Td_2_: Average decomposition temperature for the second step.

Material	Sample Group	Mass Loss at 100 °C (wt%)	Td_5_ (°C)	Td_1_ (°C)	Td_2_ (°C)
MED625FLX	Control	0.67	247 ± 8	309 ± 1	423 ± 2
	Machine/H_2_O_2_	0.66	258 ± 2	310 ± 3	422 ± 5
	Manual/H_2_O_2_	0.53	259 ± 7	307 ± 2	427 ± 5
	Machine/steam	0.8	256 ± 3	309 ± 1	423 ± 7
	Manual/steam	0.53	258 ± 2	304 ± 2	424 ± 1
Bioflex	Control	0.42	163 ± 7	326 ± 6	425 ± 3
	Machine/H_2_O_2_	1.04	240 ± 5	341 ± 3	427 ± 4
	Manual/H_2_O_2_	0.63	221 ± 6	338 ± 3	432 ± 4
	Machine/steam	0.5	252 ± 4	341 ± 2	430 ± 2
	Manual/steam	0.9	244 ± 2	340 ± 4	430 ± 4

**Table 3:** Glass transition values obtained from DSC and DMA. ^a^ The glass-transition temperatures (°C, Tg) determined from DSC analysis using the half-extrapolated tangents method of the first heating step of DSC thermogram recorded at 10 °C/min. ^b^ The glass-transition temperatures (°C, Tg) determined from DMA results from the peak maximum of the tan δ curve. Average values and standard deviations were calculated from three independent measurements for each sample.

Material	Group	Tg ^a^ (DSC) (°C)	Tg ^b^ (DMA) (°C)
MED625FLX	Control	−4 ± 2	32 ± 1
	Machine/H_2_O_2_	−1 ± 1	36 ± 1
	Manual/H_2_O_2_	−2 ± 1	36 ± 2
	Machine/steam	1 ± 1	36 ± 1
	Manual/steam	1 ± 2	35 ± 1
Bioflex	Control	−11 ± 1	22 ± 1
	Machine/H_2_O_2_	−4 ± 1	38 ± 3
	Manual/H_2_O_2_	−9 ± 1	35 ± 1
	Machine/steam	−2 ± 1	35 ± 1
	Manual/steam	0 ± 2	31 ± 2

## Data Availability

Data is contained within the article or Supplementary Material.

## Supplementary Materials

The following supporting information can be downloaded at: https://www.mdpi.com/article/10.3390/bioengineering12090924/s1, Figure S1: (A) Set-up for bending tests of specimens with 127 mm total length; (B) Design and dimensions of the small sample set in Blender; Figure S2: Micro-CT images of Bioflex specimens from the machine/steam (top) and manual/steam group (bottom) that revealed superficial cracking. Machine/steam samples show cracking with depths ranging from 0.420 mm to 0.455 mm. Manual/steam samples exhibit deeper, more extensive longitudinal cracks with depths between 0.576 mm and 0.655 mm. Subtle bulging of the material is visible in the manual/steam group; Figure S3: Load–displacement curves from three-point bending tests of MED625FLX and Bioflex (small samples); Figure S4: Exemplary dynamic mechanical analysis curves of MED625FLX for one sample per group. Storage modulus (E′, left *Y*-axis), loss modulus (E″, right *Y*-axis), and loss factor (the ratio of E″ to E′) (tan δ, right *Y*-axis) are shown. The peak in tan δ indicates Tg. Table S1: Material properties of the 3D printing resins used in this study; Table S2: Results of the three-point bending test and statistical analysis. Significant *p*_a_ values are highlighted in bold.

## Abbreviations

The following abbreviations are used in this manuscript:

3D	Three-Dimensional
CAD	Computer-Aided Design
CT	Computed Tomography
DMA	Dynamic Mechanical Analysis
DSC	Differential Scanning Calorimetry
DTG	Derivative Thermogravimetry
H_2_O_2_	Hydrogen Peroxide Sterilization
LCD	Liquid Crystal Display
MDR	Medical Device Regulations
SLA	Stereolithography
T_g_	Glass Transition Temperature
TGA	Thermogravimetric Analysis
VHP	Vaporized Hydrogen Peroxide
