# Supplementary material for: Impact of Disinfection and Sterilization on 3D-Printing Resin Performance for Surgical Guides in Cardiac Ablation Surgery
Source: Bioengineering (Basel). 2025 Aug 28;12(9):924. doi: 10.3390/bioengineering12090924 (PMC12467745; doi:10.3390/bioengineering12090924)
Supplement: Supplementary file 1 [file bioengineering-12-00924-s001.zip › bioengineering-3673895-supplementary.pdf]

SUPPLEMENTARY FIGURES

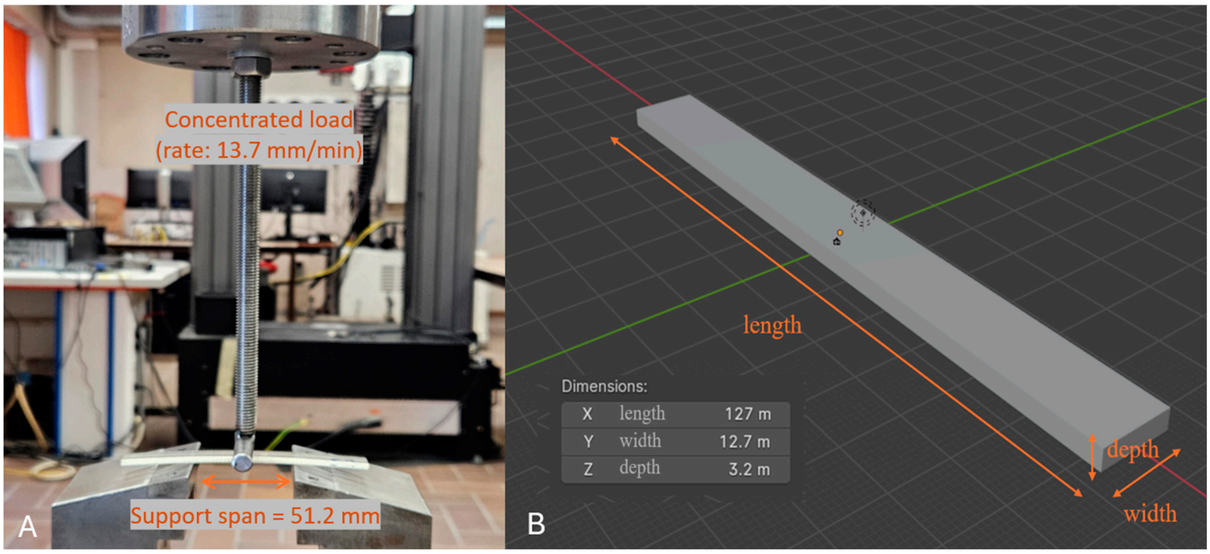

Figure S1. (A) Set-up for bending tests of specimens with 127 mm total length; (B) Design and dimensions of the small sample set in Blender.

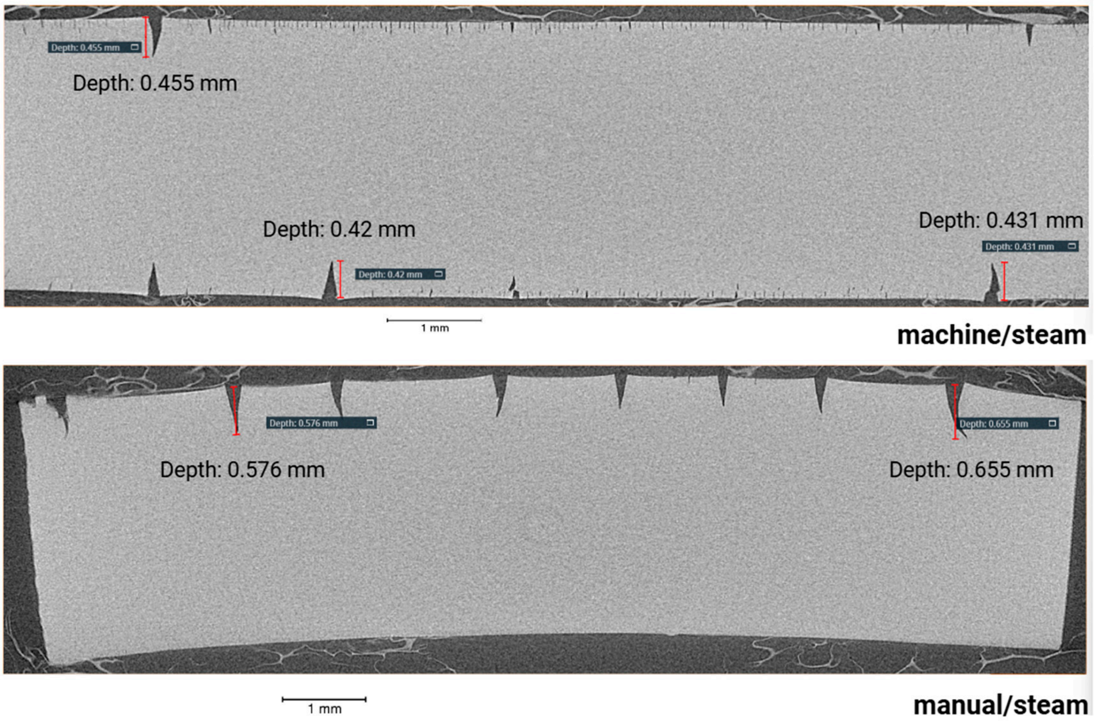

Figure S2. Micro-CT images of Bioflex specimens from the machine/steam (top) and manual/steam group (bottom) that revealed superficial cracking. Machine/steam samples show cracking with depths ranging from 0.420 mm to 0.455 mm. Manual/steam samples exhibit deeper, more extensive longitudinal cracks with depths between 0.576 mm and 0.655 mm. Subtle bulging of the material is visible in the manual/steam group.

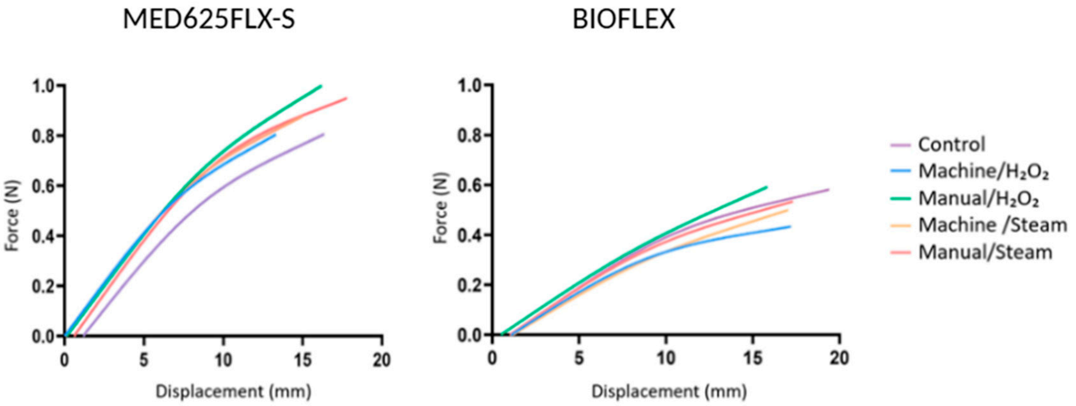

Figure S3. Load-displacement curves from three-point bending tests of MED625FLX-S and Bioflex (small samples).

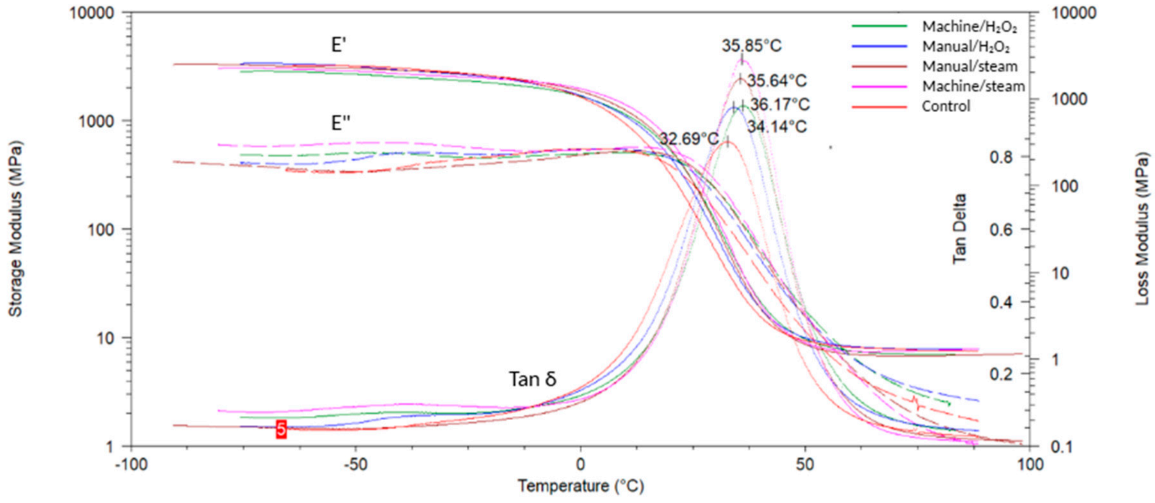

Figure S4. Exemplary dynamic mechanical analysis curves of MED625FLX for one sample per group. Storage modulus (E′, left Y-axis), loss modulus (E″, right Y-axis), and loss factor (the ratio of E″ to E′) (tan δ, right Y-axis) are shown. The peak in tan δ indicates T_g.

SUPPLEMENTAL TABLES

Table S1. Material properties of the 3D printing resins used in this study.

**Trade Name (Company)**	**Bioflex® A80 MB (3Dresyns)**	**MED625FLX® (Stratasys)**
**Chemical Family**	NP	NP
**Thermal properties:** **Glass Transition Temperature (°C) and Melting Point (°C)**	NP	NP
**Tensile Strength (MPa)**	<15	3-5 (D-412)
**Flexural strength (MPa)**	<15	NP
**Young modulus (MPa)**	<90	NP
**Elongation at Break (%)**	<100	45-55 (D-412)
**Compressive Set (%)**	N/P	0.5-1.5 (D-395)
**Shore Hardness (Scale A)**	A75-85	73-77 (D-2240)
**Tensile Tear Resistance (kg/cm)**	N/P	8-12 (D-624)
**Polymerized Density (g/cm^2)**	N/P	1.16-1.17 (D-792)
**Printer Technology**	SLA, DLP & LCD	PolyJet
**Support Removal Type**	/	WaterJet
**IZOD Impact, Notched**	N/P	20-30 J/m
**HDT heat deflection temperature (°C)**	N/P	45-50°C

**Bio-compatibility**	Approved (EN ISO 10993-1)	Approved (EN ISO 10993-1)
**Biological evaluation**	Cytotoxicity EN ISO 10993-5:2009	Cytotoxicity EN ISO 10993-5:2009<br>IrritationEN ISO 10993-10:2013<br>Delayed-type hypersensitivity EN ISO 10993-10:2013<br>Material mediated pyrogenicity (FDM USP <151>) EN ISO 10993-11:2009<br>Acute systemic toxicity EN ISO 10993-11:2009<br>Chemical characterization EN ISO 10993-18:2009<br>Allowable limits for leachable substances EN ISO 10993-17:2009<br>USP plastic class VI USP 34 <88><br>Genotoxicity EN ISO 10993-3:2014

NP: not provided

Table S2. Results of the three-point bending test and statistical analysis. Significant p_a values are highlighted in bold.

Material	Subgroup	Flexural Modulus E_f [MPa]	ANOVA		Bonferroni-Corrected Post Hoc Test (Students-t, If p<0.05)		
			F (x, x)	p	Comparison	t (x)	p_a
**MED625FLX-S**	Control	30.48 ± 0.74	3.889	0.018	machine/H_2O_2 vs manual/H_2O_2	-0.613	0.559
	Machine/ H_2O_2	30.81 ± 1.13			machine/H_2O_2 vs machine/steam	-1.657	0.139
	Manual/H_2O_2	31.21 ± 0.81			machine/H_2O_2 vs manual/steam	-2.485	0.038
	Machine/steam	31.85 ± 0.84			machine/H_2O_2 vs control	0.544	0.604
	Manual/steam	32.56 ± 1.10			manual/H_2O_2 vs machine/steam	-1.169	0.282
					manual/H_2O_2 vs manual/steam	-2.125	0.071
					manual/H_2O_2 vs control	1.385	0.213
					machine/steam vs manual/steam	-1.142	0.289
					machine/steam vs control	2.731	0.026

					manual/steam vs control	3.504	0.009
**MED625FLX-L**	Control	27.32 ± 3.88	1.837	0.198	-	-	-
	Machine/ H_2O_2	25.27 ± 0.23			-	-	-
	Manual/H_2O_2	23.57 ± 0.16			-	-	-
	Machine/steam	25.33 ± 1.44			-	-	-
	Manual/steam	23.97 ± 0.67			-	-	-
**BIOFLEX**	Control	18.86 ± 0.68	7.04	0.001	machine/H_2O_2 vs manual/H_2O_2	-2.765	0.036
	Machine/ H_2O_2	16.85 ± 0.43			machine/H_2O_2 vs machine/steam	-0.450	0.665
	Manual/H_2O_2	18.19 ± 1.00			machine/H_2O_2 vs manual/steam	-0.586	0.581
	Machine/steam	16.96 ± 0.41			machine/H_2O_2 vs control	-5.628	**0.001**
	Manual/steam	17.13 ± 0.99			manual/H_2O_2 vs machine/steam	2.538	0.049
					manual/H_2O_2 vs manual/steam	1.685	0.131

manual/H_2O_2 vs control	-1.257	0.249
machine/steam vs manual/steam	-0.341	0.746
machine/steam vs control	-5.372	**0.001**
manual/steam vs control	-3.234	0.014
